# Genome-Wide Association Study for Seed Dormancy Using Re-Sequenced Germplasm under Multiple Conditions in Rice

**DOI:** 10.3390/ijms24076117

**Published:** 2023-03-24

**Authors:** Dandan Chen, Wenli Zou, Mingpei Zhang, Jindong Liu, Liang Chen, Ting Peng, Guoyou Ye

**Affiliations:** 1 Key Laboratory of Rice Biology in Henan Province, Collaborative Innovation Center of Henan Grain Crops, College of Agronomy, Henan Agricultural University, Zhengzhou 450002, China; 2 CAAS-IRRI Joint Laboratory for Genomics-Assisted Germplasm Enhancement, Agricultural Genomics Institute at Shenzhen, Chinese Academy of Agricultural Sciences, Shenzhen 518120, China; 3 State Key Laboratory of Crop Stress Adaptation and Improvement, School of Life Sciences, Henan University, Kaifeng 475004, China; 4 Shenzhen Research Institute of Henan University, Shenzhen 518000, China; 5 Rice Breeding Innovations Platform, International Rice Research Institute (IRRI), Metro Manila 1301, Philippines

**Keywords:** seed dormancy and germination, QTLs, candidate gene, expression level, hormone

## Abstract

Seed dormancy is a key factor used to determine seed germination in rice production. So far, only a few genes controlling seed dormancy have been reported, and the genetic mechanism of rice seed dormancy is still elusive. In this study, a population of 195 diverse re-sequenced accessions from 40 countries was evaluated for the seed germination rate (GR) without dormancy breaking (WDB) as a control and under dry heating (DH) and gibberellic acid (GA) treatments, as dormancy breaking agents to identify QTLs for seed dormancy. Phenotypic assessment revealed that these accessions had abundant variations in seed dormancy. GWAS using 1,120,223 high-quality single nucleotide polymorphisms (SNPs) and a mixed linear model (MLM) incorporating both principal components (PCs) and kinship (K) identified 30 QTLs on 10 chromosomes, accounting for 7.3–20.4% of the phenotypic variance in GR. Ten of the QTLs were located in the regions of previously reported QTLs, while the rest were novel ones. Thirteen high-confidence candidate genes were predicted for the four QTLs detected in two or three conditions (*qGR4-4*, *qGR4-5*, *qGR8* and *qGR11-4*) and one QTL with a large effect (*qGR3*). These genes were highly expressed during seed development and were significantly regulated by various hormone treatments. This study provides new insights into the genetic and molecular basis of rice seed dormancy/germination. The accessions with moderate and strong dormancy and markers for the QTLs and candidate genes are useful for attaining a proper level of seed dormancy.

## 1. Introduction

Seed dormancy is an important agronomic trait related to the yield and quality of rice [1,2]. When seed dormancy is too strong, fewer and more uneven seedling emerge in seedbeds (seed raising) or in fields (direct seeding), which ultimately results in poor seedling establishment and yield formation [3,4,5]. However, when seed dormancy is too weak, pre-harvest sprouting (PHS) may occur under prolonged rainfall (high humidity) and high-temperature conditions, resulting in the loss of quality and yield [6,7]. It is estimated that the annual economic losses caused by PHS total one billion U.S. dollars globally [8,9]. Rice is the primary and stable food source for more than half of the world’s population [10]. Therefore, developing varieties with moderate seed dormancy is an important objective of rice breeding. To accelerate breeding by marker-assisted selection, markers closely linked to QTLs or functional markers for genes with natural variants are essential [11]. Therefore, it is important to identify and characterize genes controlling seed dormancy and to elucidate the genetic mechanisms underlying seed dormancy in rice. 

Numerous studies have demonstrated that the seed dormancy is influenced by many factors, including internal (embryo, seed coat, and endogenous inhibitors) [12] and environmental factors (temperature, light and air, etc.) [13,14,15]. In rice production practice, it is necessary to break seed dormancy in order to improve the germination rate (GR) and seedling neatness, and the most commonly used methods are dry heating (DH) and gibberellic acid (GA) treatments. DH changes the seed coat structure and increases the permeability of the seed shell and air permeability, which promotes the seed metabolism and stimulates seed germination and seedling growth [16,17]. GA immersion encourages tissue weakening around the embryo, overcomes the bonding of the seed coat to the seed embryo and promotes seed embryo growth [16,18]. 

Seed dormancy is a complex quantitative trait controlled by multiple genes [19,20,21,22]. To date, hundreds of QTLs associated with seed dormancy in rice have been reported, mainly through linkage mapping using biparental populations in cultivated, wild and weedy rice. The QTL mapping of other traits in rice populations including recombinant inbred lines (RILs), F_2_ and chromosome segment substitution lines (CSSLs) is commonly used [23,24,25,26,27,28,29]. Among them, a few QTLs have been finely mapped, such as *Sdr1* [30], *qLTG3-1* [26], *qSD12* [31], *qSDn-1* [20], *qSDn-5* [20], *qSD7-1* [27], *qSD10* [32], *qDOM3.1* [28], *qSdr9* [33] and *qSD6* [22]. Genome-wide association studies (GWAS) based on linkage disequilibrium (LD) is a powerful method to identify marker-trait associations (MTAs) without creating artificial mapping populations and has been used in dissecting the genetic basis of complex traits including seed dormancy. A GWAS using 350 rice varieties found worldwide detected 16 and 38 QTLs significantly associated with GR in newly harvested and late-ripening seeds, respectively, with *FHS1.1*, *FHS11* and *FHS5.2* being identified using both the newly harvested and late maturing seeds [34]. Another GWAS using 453 *indica* rice accessions identified nine QTLs for panicle GR on 8 chromosomes [2]. A more recent study using 311 accessions identified eight QTLs for seed dormancy measured by the germination index (GI) [35]. 

The lack of functional genes with natural variations has been a major obstacle to marker-assisted breeding for proper seed dormancy in rice. To date, only five genes with natural variations have been functionally characterized. The first map-based cloned gene is *Sdr4* in the *qSD12* region [36]. *Sdr4* is positively regulated by the seed maturation regulatory gene Osviviparous1 (*OsVP1*) and positively regulates three seed maturation-related protein genes *OsDOG1-Likes* and suppresses several germination-related genes, including a gibberellin biosynthesis gene (*OsGA20ox-1*), two aquaporin genes (*PIP1;3* and *PIP2;2*) and an expansion gene (*OsEXPB3*) after germination, thus acting as a mediator of seed dormancy during seed maturation [36]. The interaction between qSD7-1/Rc, a MYB transcriptional activator (OsC1-Myb), and OsVP1 enhances seed sensitivity to abscisic acid (ABA) by promoting proanthocyanidin (PA) biosynthesis and the perception of ABA signals, and this ultimately inhibits the PHS [37]. Naturally occurring or induced loss-of-function mutations of the gibberellic acid (GA) synthesis gene (*qSD1-2/OsGA20ox2*) enhance seed dormancy and reduce the plant’s height, probably through the GA-induced dehydration mechanism [38]. OsDOG1L-3 with homology to *Arabidopsis* DOG1 has recently been reported to be the functional gene for the seed dormancy QTL, *qSd-1-1*, identified in a population derived from N22 and Nanjing35 [17,37]. *OsDOG1L-3* increases the ABA content by up-regulating the expression of several ABA-related genes [17,37]. OsbZIP75 and OsbZIP78 directly bind to the ABRE element in the promoter region of *OsDOG1L-3* to promote its expression [17]. *SD6* encoding a basic helix-loop-helix (bHLH) transcription factor (*OsbHLH048*) contributes to the natural variation of seed dormancy [32]. *SD6* and the inducer of C-repeat binding factors expression 2 (*ICE2*) function antagonistically in controlling seed dormancy by directly regulating the ABA catabolism gene *ABA8OX3*, and indirectly regulating the ABA biosynthesis gene *NCED2* [34]. In this study, to identify novel QTLs and candidate genes for seed dormancy, we measured the seed GR of 195 rice accessions with high-quality sequence information under three treatments, including one without dormancy breaking (WDB) as a control and DH and GA treatments as dormancy breaking agents, and performed GWAS analysis. By combining information from genome annotation, genomic variations, reported functions and public expression databases, candidate genes were suggested for five important QTLs. Our study provided useful information for deciphering the genetic mechanism of rice seed dormancy/germination and molecular breeding.

## 2. Results

### 2.1. Phenotypic Analysis of Seed Dormancy

**Figure 1:**
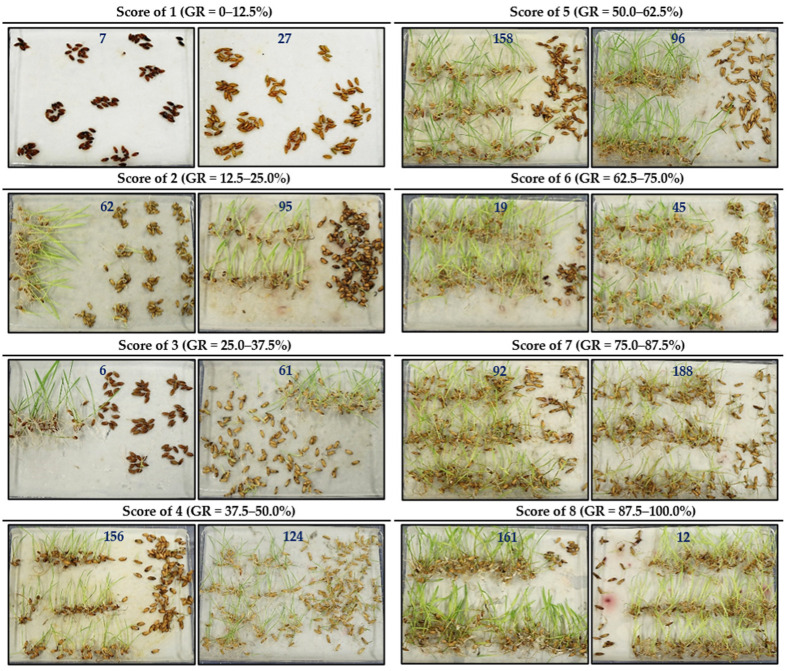
Representative images of accessions with different seed dormancy scores. 1, germination rate (GR) 0–12.5%. 2, GR 12.5–25%. 3, GR 25–37.5%. 4, GR 37.5–50%. 5, GR 50–62.5%. 6, GR 62.5–75%. 7, GR 75–87.5% and 8, GR 87.5–100% [39]. Two accessions are shown for each seed dormancy score, and the numbers are the accession codes.

**Figure 2:**
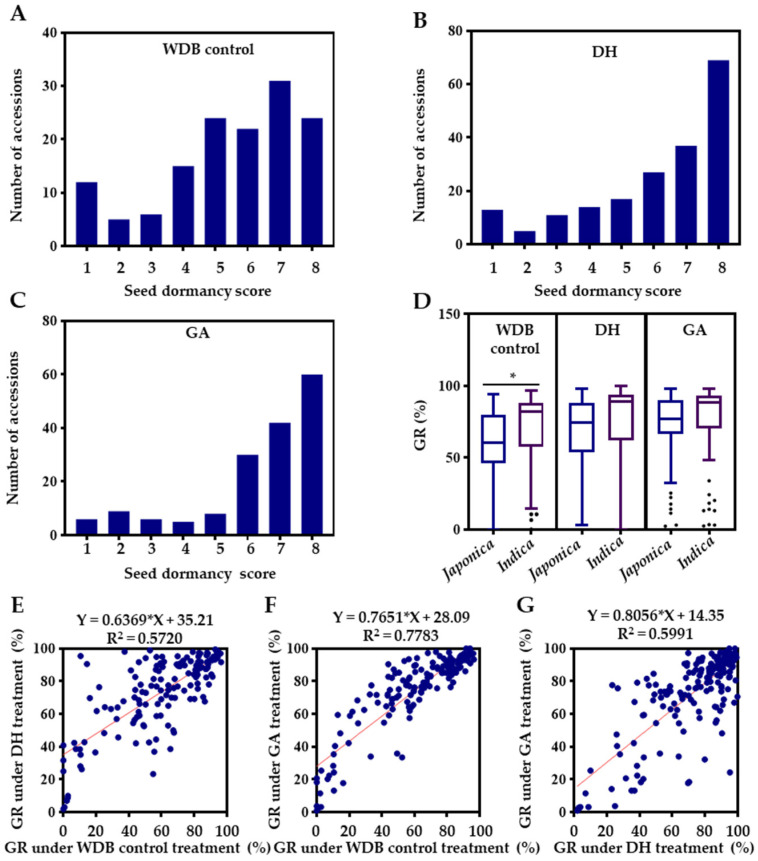
Distribution of seed dormancy scores and correlation between treatments. Distribution under without dormancy breaking (WDB) as a control treatment (**A**), dry heating (DH) (**B**) and gibberellic acid (GA) treatments as dormancy breaking agents (**C**). (**D**) Comparison of GR between *japonica* and *indica* subspecies. (**E**–**G**) Correlation between WDB control and DH (**E**), WDB control and GA (**F**), and DH and GA (**G**) treatment. “*” indicates significance at *p* ≤ 0.05.

A rice diversity panel of 195 diverse rice accessions collected from 40 countries by the International Rice Research Institute (IRRI), representing the major rice-growing regions in the world, was used to evaluate seed dormancy under three conditions (Table S1). Seed GR, the most commonly used criterion, was used to measure seed dormancy [18,19]. The GR had ranges of 0–96.7%, 0–100.0% and 2.4–98.5% under WDB control, DH and GA treatments, respectively (Table 1), indicating that a wide variation in seed dormancy exists in these accessions. As expected, the variation was reduced by the dormancy breaking treatments (DH and GA). Based on the previously described seed dormancy scoring method [39], 195 accessions were grouped into 8 categories (Figure 1). The GR showed a skewed distribution toward high GR under all the three treatments (Figure 2A–C). There were only small proportions of the accessions with deep dormancy (scores 1, 2 and 3), which were 16.5%, 15.0% and 12.7% for the WDB control, DH and GA treatments, respectively, while the proportions of non-deep dormancy (scores 7 and 8) accessions were higher, at 39.6%, 54.9% and 61.5%, respectively (Figure 2A–C). This was expected since strong dormancy is undesirable for rice production and is negatively selected during domestication and breeding. Therefore, seed dormancy is greatly reduced in modern rice varieties to meet production needs [40]. The accessions with strong dormancy can be used to identify novel genes. The average GRs under the WDB control, DH and GA treatments were 62.4%, 69.0% and 73.6%, respectively (Table 1). The DH and GA treatments had significantly higher GRs than that of the WDB control (Table 1), indicating that the DH and GA treatments were effective at promoting seed germination. The mean GR under the GA treatment was higher than that of the DH treatment (Table 1), indicating that the GA treatment is more effective in breaking dormancy than the DH treatment is. 

The average GR of the *indica* accessions was significantly higher than that of the *japonica* accessions under the WDB control, while the difference between the two subspecies was not significant under the DH and GA treatments (Figure 2D), although *indica* had a slightly higher GR than *japonica* did (Figure 2D, Table 1). 

The correlations between the three treatments were positive and strong, with the correlation coefficients being 0.71, 0.88, and 0.74 for WDB control and DH, WDB control and GA, and DH and GA, respectively (Figure 2E–G).

### 2.2. Single Nucleotide Polymorphism (SNP) Data, Population Structure and LD Pattern in the Panel

**Figure 3:**
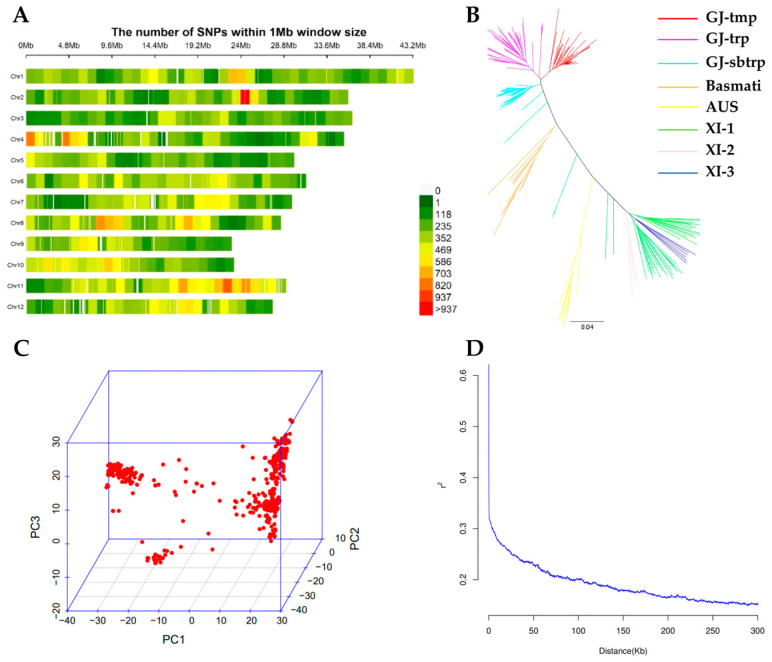
Genomic distribution of single nucleotide polymorphism (SNP) markers and population structure and linkage disequilibrium (LD) analysis of the population of 195 rice accessions. (**A**) Distribution of SNP markers used in GWAS. (**B**) Neighbor-joining (NJ) tree. GJ, Geng/Japonica. XI, Xian/Indica. tmp, temperate. trp, tropical. sbtrp, subtropical. (**C**) Principal component analysis (PCA) plots. (**D**) LD decay analysis.

The sequencing data were mapped to the Nipponbare reference genome (IRGSP 1.0). After removing the SNPs with a minor allele frequency (MAF) ≤ 5% or a missing data rate ≥ 10%, 1,120,223 SNPs were left and employed for the GWAS (Figure 3A). The chromosome size ranged from 22.8 Mb for chromosome 9 to 43.2 Mb for chromosome 1 (Figure 3A). These markers spanned a physical distance of 373 Mb, with an average density of 3 markers per kb (Figure 3A). The neighbor-joining (NJ) tree showed eight branches representing the eight subpopulations (Figure 3B), indicating that the panel has a good coverage of the genetic diversity of cultivated rice and that the population structure must be properly handled in an association analysis. The first three principal components (PCs) explained 16.2%, 6.9% and 3.8% of the total variation, respectively, and clearly separated the two subspecies (Figure 3C), suggesting that the major population structure could be corrected in association analysis by incorporating the top few PCs. LD decay along with physical distances was computed for the whole panel. The scatter *r^2^* against the physical distance showed a clean pattern of LD decay (Figure 3D). The LD decay distance was approximately 100 kb (Figure 3D) based on the threshold determined by the *r^2^* value at the 95th percentile its intersection with the LD decay curve. 

### 2.3. GWAS of Seed Dormancy

**Figure 4:**
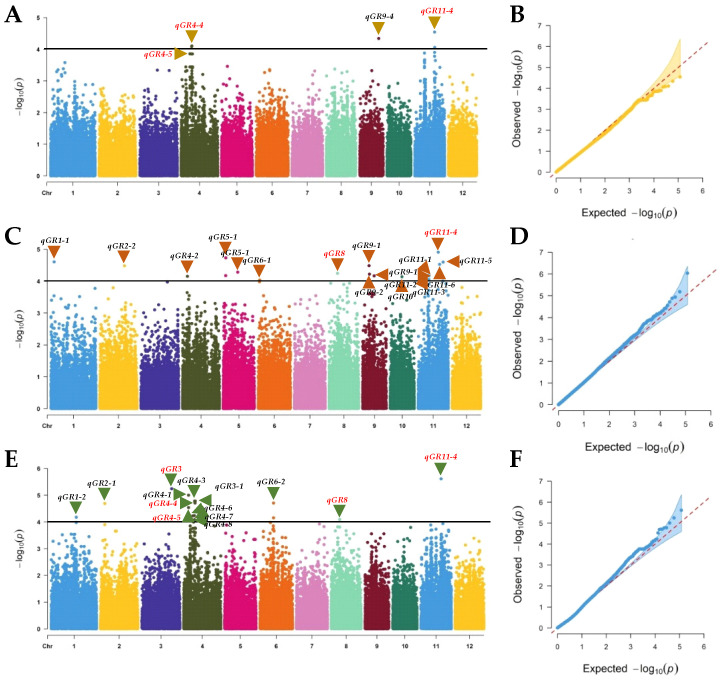
Genome-wide association studies of seed dormancy measured as germination rate (GR) using a population of 195 diverse rice accessions. Manhattan plots and Quantile–Quantile (QQ) plots for GRs in the without dormancy breaking (WDB) control (**A**,**B**), dry heating (DH) treatment (**C**,**D**) and gibberellic acid (GA) treatment (**E**,**F**). The black horizontal lines, indicate the genome-wide significance threshold (*p* = 10^−4^). The names of QTLs are given above the arrows pointing to the peak markers.

**Figure 5:**
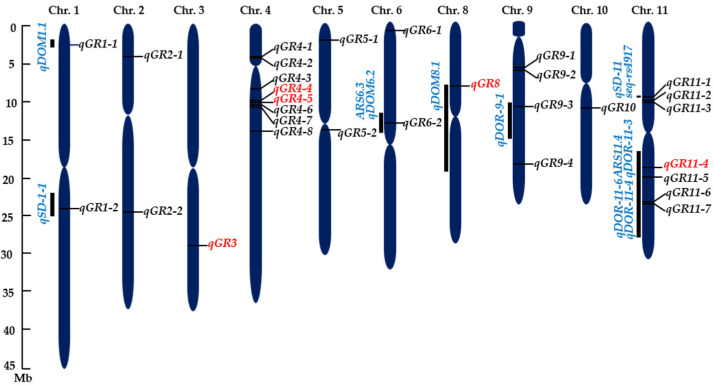
Distribution of QTLs for seed dormancy measured as germination rates (GR) identified using GWAS in a rice diversity panel. QTLs in red are the ones chosen for candidate gene prediction. QTLs in blue have been previously reported. Distances are in Mb.

The associations between the SNP markers and GR were identified for the three treatments separately using a mixed linear model (MLM) that controlled the population structure through principal component analysis (PCA) and cryptical genetic relatedness through kinship coefficients (K). The results are presented as Manhattan and Quantile–Quantile (QQ) plots (Figure 4). A marker was considered significantly associated with the trait when *p* ≤ 10^−4^. We used a relatively more liberal threshold *p* value based on the following considerations. First, we wanted to reduce the possible false negatives due to possible overcorrection by the analytical model. Second, the detection power is likely to be low, particularly for markers with lower minor allele frequency due to the relatively small population size. Third, other sources of information can be used to evaluate the reliability of the putative QTLs identified. Five, nineteen and sixteen MTAs were found in the WDB control, DH and GA groups, respectively (Table S2). These MTAs were delimited into 30 QTLs by regarding the adjacent SNPs in 200 kb (±100 kb) of a peak SNP as a single QTL (Figure 4 and Figure 5, Table 2). These QTLs explained 7.3–20.4% of the phenotypic variance.

The *qGR4-4* and *qGR4-5* were identified under the WDB control and GA treatments. *qGR4-4* on chromosome 4 (10.0–10.2 Mb) explained 14.8% and 17.6% of the phenotype variation, respectively (Table 2), while *qGR4-5* on chromosome 4 (10.3–10.5 Mb) explained 16.9% and 20.4% of the phenotype variation, respectively (Table 2). *qGR8* on chromosome 8 (7.9–8.2 Mb) was found under the DH and GA treatments and explained 8.9% and 14.3% of the phenotypic variation, respectively (Table 2). *qGR11-4*, located on chromosome 11 (19.2–19.4 Mb), was identified under all three treatments, which explained 16.3%, 10.2% and 20.2% of the trait variation under the WDB control, DH and GA treatments, respectively (Table 2). Although *qGR3* (28.4–28.6 Mb) was found only under the GA treatment, it was the strongest QTL detected in a single treatment, explaining 18.7% of the phenotypic variation (Table 2). 

**Table 2:** QTLs identified for seed dormancy measured as germination rates (GR) under three different treatments using a rice diversity panel.

QTL	Treatment	Chr.	Peak SNP	Effect	SE	*p*-Value	*R* ^2^	Germination Indicator Used in Previous Study	References
*qGR1-1*	DH	1	2,998,209	1.0	3.8	2.49 × 10^−5^	10.8%	T50 (time to reach 50% germination of the total number of germinated seeds). U8416 (time interval between 84% and 16% of viable seed to germinate). Intact seed harvested on 35 day after flowering.	[28]
*qGR1-2*	GA	1	23,870,065	18.4	5.3	6.68 × 10^−5^	16.8%	GR of intact seed (35 day after heading (DAH)) at day seven.	[41]
*qGR2-1*	GA	2	4,193,857	23.8	6.5	2.04 × 10^−5^	13.8%	-	-
*qGR2-2*	DH	2	23,728,701	−15.5	5.9	3.31 × 10^−5^	9.3%	-	-
*qGR3*	GA	3	28,515,163	−24.0	6.8	5.69 × 10^−5^	18.7%	-	-
*qGR4-1*	GA	4	4,523,405	19.3	5.5	1.00 × 10^−5^	17.8%	-	-
*qGR4-2*	DH	4	4,662,452	17.2	6.8	7.07 × 10^−5^	8.7%	-	-
*qGR4-3*	GA	4	8,563,180	−18.3	6.4	5.97 × 10^−5^	14.8%	-	-
*qGR4-4*	WDB control	4	10,123,838	27.7	7.1	8.32 × 10^−5^	17.5%	-	-
	GA	4	10,123,838	27.7	7.1	5.79 × 10^−5^	14.8%	-	-
*qGR4-5*	GA	4	10,352,980	−24.0	9.1	1.66 × 10^−5^	16.9%	-	-
	WDB control	4	10,355,218	20.6	7.3	7.79 × 10^−5^	20.4%	-	-
*qGR4-6*	GA	4	10,726,116	−23.8	7.2	1.95 × 10^−5^	16.6%	-	-
*qGR4-7*	GA	4	10,981,781	24.0	9.1	1.66 × 10^−5^	16.9%	-	-
*qGR4-8*	GA	4	14,056,148	6.6	6.6	7.05 × 10^−5^	14.5%	-	-
*qGR5-1*	DH	5	2,322,980	1.0	5.1	1.85 × 10^−5^	9.8%	-	-
*qGR5-2*	DH	5	13,914,542	7.7	4.1	5.21 × 10^−5^	8.9%	-	-
*qGR6-1*	DH	6	1,484,749	3.8	3.6	9.29 × 10^−5^	9.6%	-	-
*qGR6-2*	GA	6	13,120,454	27.6	7.2	1.95 × 10^−5^	16.6%	GR at day seven. GR at day three. T50. U8416. AUC (area under the germination curve). GI (germination index). GR of after-ripened intact seed (32 DAH) at day seven.	[28,34]
*qGR8*	DH	8	8,040,564	24.5	11.6	5.68 × 10^−5^	8.9%	GR at day three. T50.	[28]
	GA	8	8,073,758	−30.7	7.5	8.14 × 10^−5^	14.3%		
*qGR9-1*	DH	9	6,327,905	−29.9	9.5	3.27 × 10^−5^	10.5%	-	-
*qGR9-2*	DH	9	6,672,606	5.2	5.2	6.01 × 10^−5^	8.8%	-	-
*qGR9-3*	DH	9	11,261,867	10.2	10.6	6.80 × 10^−5^	8.7%	GR of intact seed and de-hulled seed (30 DAH) at day seven.	[42]
*qGR9-4*	WDB control	9	18,641,166	−24.8	7.3	4.53 × 10^−5^	15.4%	-	-
*qGR10*	DH	10	11,242,705	0.7	4.5	7.28 × 10^−5^	8.6%	-	-
*qGR11-1*	DH	11	9,730,691	−3.0	7.6	6.57 × 10^−5^	7.3%	GR of intact seed (30–40 DAH) at ten days. GR of intact seed (effective cumulative temperature reached 600° after heading) at day seven.	[2,29]
*qGR11-2*	DH	11	10,370,801	−9.4	5.1	8.89 × 10^−5^	9.6%	-	-
*qGR11-3*	DH	11	10,544,254	10.2	5.6	6.75 × 10^−5^	9.9%	-	-
*qGR11-4*	WDB control	11	19,263,268	−23.5	9.0	2.84 × 10^−5^	16.3%	GR of after-ripened intact seed (32 DAH) at day seven. GR of intact seed (30 DAH) at day seven.	[34,42]
	DH	11	19,313,112	−6.4	6.6	1.23 × 10^−5^	10.1%		
	GA	11	19,313,112	−6.4	6.6	2.44 × 10^−5^	20.2%		
*qGR11-5*	DH	11	20,619,141	19.9	9.1	2.97 × 10^−5^	9.4%	GR of de-hulled seed (60 DAH) at day seven.	[42]
*qGR11-6*	DH	11	23,872,614	4.6	4.4	9.96 × 10^−5^	9.5%	GR of intact seed (30 and 60 DAH) at day seven.	[42]
*qGR11-7*	DH	11	24,096,053	−1.9	3.8	2.52 × 10^−5^	10.7%	GR of intact seed (30 and 60 DAH) at day seven.	[42]

WDB control, without dormancy breaking as a control treatment. DH, dry heating treatment. GA, gibberellic acid treatment. SE, standard error.

### 2.4. Candidate Gene Prediction

In the regions of the five QTLs, including *qGR3*, *qGR4-4*, *qGR4-5*, *qGR8* and *qG11-4*, there are 163 annotated genes in the rice annotation project database (https://rapdb.dna.affrc.go.jp/, accessed on 1 October 2022) (Table S3). After removing the genes annotated as expressed, hypothetical, transposon and retrotransposon proteins, 43 genes remained (Table S4). Among them, 20 genes potentially have functions in seed dormancy/germination based on the gene annotation information (Table 3). Out of the 20 genes, 15 genes had non-synonymous mutations in the CDS coding regions and mutations in the 2000 bp of the promoter regions, and the other 5 genes had mutations only in the promoter regions in the 3K sequencing population (Rice SNP-Seek database, https://snp-seek.irri.org/, accessed on 25 November 2022, and RFGB database, https://www.rmbreeding.cn/, accessed on 25 November 2022) (Table S5). The seven candidate genes for *qGR3* were *LOC_Os03g49990*, *LOC_Os03g50050*, *LOC_Os03g50110*, *LOC_Os03g50120*, *LOC_Os03g50130*, *LOC_Os03g50140* and *LOC_Os03g50160* (Table 3). The two candidate genes for *qGR4-4* were *LOC_Os04g18200* and *LOC_Os04g18380,* and the two for *qGR4-5* were *LOC_Os04g18650* and *LOC_Os04g18790*. The three candidate genes for *qGR8* were *LOC_Os08g13360*, *LOC_Os08g13440* and *LOC_Os08g13640*. The six candidate genes for *qGR11-4* were *LOC_Os11g32510*, *LOC_Os11g32610*, *LOC_Os11g32650*, *LOC_Os11g32720*, *LOC_Os11g32750* and *LOC_Os11g32810* (Table 3). Based on functional analysis of genes/homologs reported in the literature, seven of the genes, including *LOC_Os03g50050*, *LOC_Os03g50130*, *LOC_Os04g18380*, *LOC_Os04g18650*, *LOC_Os04g18790*, *LOC_Os11g32750* and *LOC_Os11g32810,* were considered as high-confidence genes, since they are likely to play important roles in the synthesis and metabolism of GA, ABA or other hormones.

We further analyzed the expression of the 20 candidate genes in embryos during the seed imbibition stage using a dataset downloaded from the European Bioinformatics Institute (EBI) database (https://www.ebi.ac.uk/, accessed on 1 November 2022) [43]. Except for *LOC_Os03g49990*, *LOC_Os04g18380* and *LOC_Os04g18790* that were not available in the data, all 17 genes were differentially up- or down-regulated during seed imbibition (Table 3). Only eight genes were significantly regulated during imbibition with at least a two-fold change (Table 3). *LOC_Os03g50140*, *LOC_Os03g50160* and *LOC_Os11g32750* were up-regulated, while *LOC_Os03g50110*, *LOC_Os04g18200* and *LOC_Os08g13640* were down-regulated after imbibition for 12 h and 24 h (Table 3). The expression of *LOC_Os11g32650* was up-regulated only after 12 h of imbibition, while that of *LOC_Os03g50130* was significantly up-regulated only after 24 h of imbibition (Table 3). These eight genes were also considered as high-confidence candidate genes associated with seed dormancy/germination. 

By combining these eight genes selected by EBI database and the seven genes selected by literate information, a total of thirteen high-confidence candidate genes were finally predicted for the five QTLs. They were *LOC_Os03g50050* encoding an F-box and DUF domain-containing protein, *LOC_Os03g50110* encoding a GEBP transcription factor (Fragment), *LOC_Os03g50130* encoding the microsomal glutathione S-transferase 3, *LOC_Os03g50140* and *LOC_Os03g50160* encoding plastocyanin-like domain containing proteins, *LOC_Os04g18200* encoding dihydrodipicolinate synthase (chloroplast precursor), *LOC_Os04g18380* encoding a cytochrome P450 enzyme, *LOC_Os04g18650* encoding an Apetala2 (AP2) domain-containing protein, *LOC_Os04g18790* encoding an F-box domain-containing protein, *LOC_Os08g13640* encoding an Ankyrin protein, *LOC_Os11g32650* encoding the chalcone synthase (CHS), *LOC_Os11g32750* encoding a NUDIX family hydrolase and *LOC_Os11g32810* encoding a leucine rich repeat (LRR) family protein.

**Table 3:** Candidate genes for five QTLs for seed dormancy measured as germination rate (GR).

QTL	Candidate Genes	Chr.	Functional Annotation	log_2_(12 h/0 h) *	log_2_(24 h/0 h) *	References
*qGR3*	*LOC_Os03g49990*	3	GRAS family transcription factor domain containing protein, expressed	0.4	NA	-
	*LOC_Os03g50050*	3	OsFBDUF16-F-box and DUF domain containing protein, expressed	−0.4	−0.5	[44,45,46,47]
	*LOC_Os03g50110*	3	Similar to GEBP transcription factor (Fragment)	−1.9	−1.9	[48,49,50,51,52]
	*LOC_Os03g50120*	3	Zinc finger family protein, putative, expressed	−0.4	−0.9	-
	*LOC_Os03g50130*	3	Microsomal glutathione S-transferase 3, putative, expressed	NA	1	[53,54,55,56]
	*LOC_Os03g50140*	3	Plastocyanin-like domain containing protein, putative, expressed	4.3	4.2	[57]
	*LOC_Os03g50160*	3	Plastocyanin-like domain containing protein, putative, expressed	4.5	3.7	[57]
*qGR4-4*	*LOC_Os04g18200*	4	Dihydrodipicolinate synthase, chloroplast precursor, putative, expressed	−1.2	−1.2	[58,59,60,61]
	*LOC_Os04g18380*	4	Cytochrome P450, putative, expressed	NA	NA	[62,63]
*qGR4-5*	*LOC_Os04g18650*	4	AP2 domain containing protein, expressed	−0.4	−0.3	[64,65,66]
	*LOC_Os04g18790*	4	OsFBX126-F-box domain containing protein, expressed	NA	NA	[44,45,46,47]
*qGR8*	*LOC_Os08g13360*	8	Kelch repeat protein, putative, expressed	−0.5	−0.4	-
	*LOC_Os08g13440*	8	Cupin domain containing protein, expressed	NA	0.7	-
	*LOC_Os08g13640*	8	Ankyrin, putative, expressed	−0.9	−1	[67,68]
*qGR11-4*	*LOC_Os11g32510*	11	OsGH3-13-Auxin-responsive GH3 gene family member, expressed	NA	0.6	-
	*LOC_Os11g32610*	11	Chalcone and stilbene synthases, putative, expressed	NA	NA	-
	*LOC_Os11g32650*	11	Chalcone synthase, putative, expressed	3.1	NA	[69,70,71,72]
	*LOC_Os11g32720*	11	Zinc knuckle domain containing protein, expressed	NA	−0.4	-
	*LOC_Os11g32750*	11	Hydrolase, NUDIX family, domain containing protein, expressed	1.3	1.8	[73,74]
	*LOC_Os11g32810*	11	Leucine Rich Repeat family protein, expressed	−0.3	−0.8	[75,76,77]

* log_2_ fold change of the expression in the seeds after 12 h and 24 h of imbibition relative to that of in the dry seeds (0 h). NA indicates data are not available. The differences in expression rates for all genes are significant at *p* < 0.05.

### 2.5. Expression Pattern throughout the Growth Period and Hormone Response of Candidate Genes

**Figure 6:**
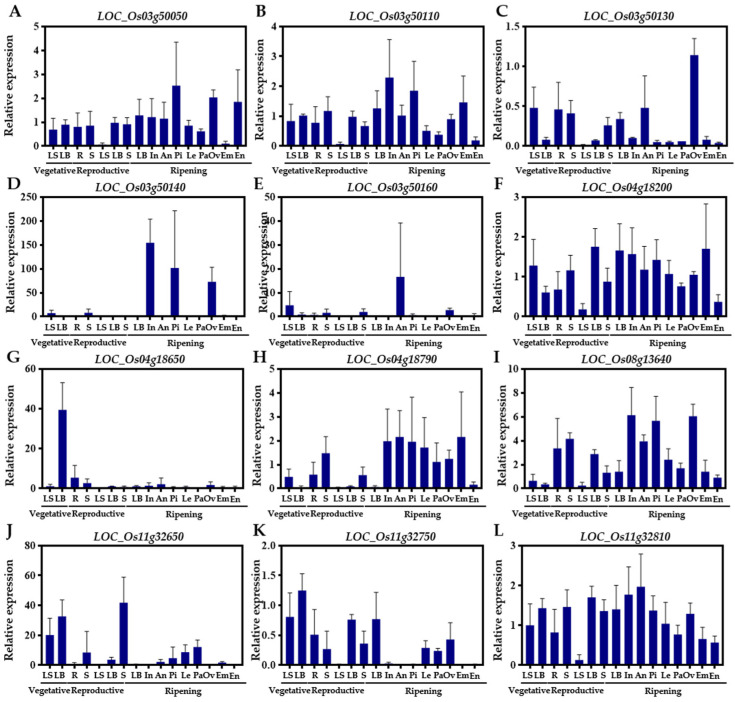
Expression profiles of high-confident candidate genes in different tissues/organs through whole growth period. Data were downloaded from RiceXPro (https://ricexpro.dna.affrc.go.jp, accessed on 1 November 2022) [78]. (**A**) *LOC_Os03g50050*, (**B**) *LOC_Os03g50110*, (**C**) *LOC_Os03g50130*, (**D**) *LOC_Os03g50140*, (**E**) *LOC_Os03g50160*, (**F**) *LOC_Os04g18200*, (**G**) *LOC_Os04g18650*, (**H**) *LOC_Os04g18790*, (**I**) *LOC_Os08g13640*, (**J**) *LOC_Os11g32650*, (**K**) *LOC_Os11g32750* and (**L**) *LOC_Os11g32810*.R, Root. LS, Leaf sheath. LB, Leaf blade. S, Stem. In, Inflorescence (3.0–4.0 mm). An, Anther (1.6–2.0 mm). Pi, Pistil (14–18 mm). Le, Lemma (7.0 mm). Pa, Palea (7.0 mm). Ov, Ovary (1 days after flowering). Em, Embryo (14 days after flowering). En, Endosperm (14 days after flowering). The expression level in roots at the vegetative stage was used as the control for analysis. Values are the mean ± SD of three independent replicates.

Since previous studies have shown that seed dormancy-related functional genes are expressed mainly in the embryos, endosperms, inflorescences and seeds [3,36,38], we used RiceXPro (https://ricexpro.dna.affrc.go.jp, accessed on 1 November 2022) [78] to evaluate the expression patterns in different tissues/organs throughout the whole growth period of 12 predicted candidate genes (*LOC_Os04g18380* was not available in the database). *LOC_Os03g50050* constitutively expressed the highest expression levels in the ovaries and endosperms (Figure 6A). *LOC_Os03g50110* expressed, in all tissues, the highest expression levels in the inflorescences and embryos (Figure 6B). *LOC_Os03g50130* and *LOC_O03g50160* strongly expressed in the ovaries (Figure 6C,E). *LOC_Os03g50140* expressed mainly during ripening, with high expression levels in the inflorescences and ovaries (Figure 6D). *LOC_Os04g18200* had higher expression levels in the leaves at the vegetative, reproductive and ripening stages and in the inflorescences, ovaries and embryos (Figure 6F). *LOC_Os04g18650* had a high expression level in the vegetative leaves and a low expression level in the inflorescences and ovaries (Figure 6G). *LOC_Os04g18790* was highly expressed in the inflorescences, ovaries and embryos (Figure 6H). *LOC_Os08g13640* was expressed mainly during ripening, particularly in the inflorescences and ovaries (Figure 6I). *LOC_Os11g32650* was highly expressed in all the tissues/organs, except the embryos (Figure 6J). *LOC_Os11g32750* was expressed in the vegetative, reproductive and ripening tissues/organs, but the expression levels were low (Figure 6K). *LOC_Os11g32810* was constitutively and highly expressed in all the tissues/organs (Figure 6L). 

**Figure 7:**
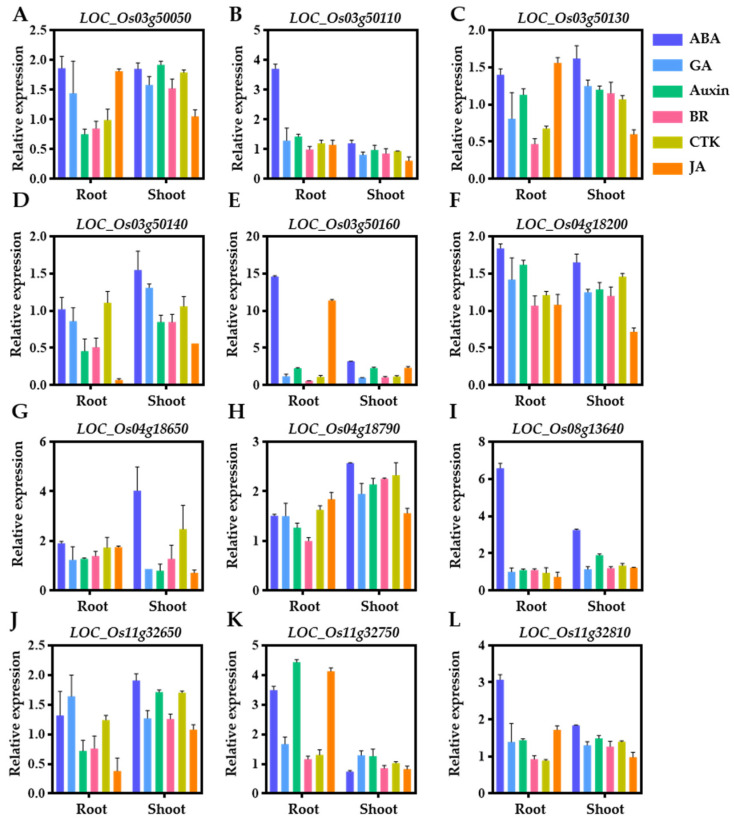
Responses of high-confidence candidate genes for seed dormancy QTLs to hormone treatments in roots and shoots of rice seedlings. Data were obtained from RiceXPro (https://ricexpro.dna.affrc.go.jp, accessed on 1 November 2022) [78]. (**A**) *LOC_Os03g50050*, (**B**) *LOC_Os03g50110*, (**C**) *LOC_Os03g50130*, (**D**) *LOC_Os03g50140*, (**E**) *LOC_Os03g50160*, (**F**) *LOC_Os04g18200*, (**G**) *LOC_Os04g18650*, (**H**) *LOC_Os04g18790*, (**I**) *LOC_Os08g13640*, (**J**) *LOC_Os11g32650*, (**K**) *LOC_Os11g32750* and (**L**) *LOC_Os11g32810*. Root and shoot samples of 7-day-old seedlings were collected at 3 h of incubation after hormone (ABA, GA, Auxin, BR, CTK and JA) treatments. The expression level in untreated seedlings (0 h) was used as the control for analysis. Values are the mean ± SD of three independent replicates.

Phytohormones play a crucial role in controlling seed dormancy/germination by regulating the expression of many genes through various signal transduction pathways [1,79], which might be a highly conserved mechanism among seed plants. To investigate the responses of the predicated candidate genes to plant hormones, their expression levels in the roots and shoots under different hormone treatments (ABA, GA, Auxin, brassinolide (BR), cytokinin (CTK) and jasmonic acid (JA)) were analyzed using the data from a curated study downloaded from the RiceXPro database (https://ricexpro.dna.affrc.go.jp, accessed on 1 November 2022, [78]). The expression of *LOC_Os04g18380* was not available. The expression of *LOC_Os03g50050* in the roots was significantly up-regulated by the ABA, GA and JA treatments, but down-regulated by the Auxin and BR treatments, while in shoots, it was up-regulated by all the six tested hormones treatments, except JA (Figure 7A). *LOC_Os03g50110* was up-regulated in roots by all the hormone treatments, except BR, while in shoots it was up-regulated by ABA, but down-regulated by the GA and JA treatments (Figure 7B). *LOC_Os03g50130* was down-regulated in roots by the GA, BR and CTK treatments, but up-regulated by the ABA, Auxin and JA treatments, while it was up-regulated by all the six tested hormones, except JA, in the shoots. (Figure 7C). A significantly down-regulated expression of *LOC_O03g50140* was observed in both the roots and shoots treated with the Auxin, BR and JA treatments, while a significantly up-regulated expression was found in shoots treated with ABA and GA (Figure 7D). The expression of *LOC_Os03g50160* was strongly up-regulated in the roots and shoots by the ABA, Auxin, CTK and JA treatments, while it was significantly down-regulated in the roots by the BR treatment (Figure 7E). *LOC_Os04g18200* was down-regulated by JA, but up-regulated by the ABA, GA, Auxin, BR and CTK treatments in the shoots, while it was up-regulated by the ABA, GA, Auxin and CTK treatments in the roots (Figure 7F). The expression of *LOC_Os04g18650* in roots was up-regulated by all the hormone treatments, while in the shoots, it was up-regulated by the ABA, BR and CTK treatments, but down-regulated by the GA, Auxin and JA treatments (Figure 7G). Significant up-regulations of *LOC_Os04g18790* were observed in both the roots and shoots by all the hormone treatments except BR, and *LOC_Os04g18790* was up-regulated by the BR treatment only in the shoots (Figure 7H). *LOC_Os08g13640* was significantly up-regulated by the ABA treatment and down-regulated by the JA treatment in the roots, while it was significantly up-regulated in the shoots by all the hormone treatments (Figure 7I). The expression of *LOC_Os11g32650* was significantly up-regulated by the ABA, GA and CTK treatments and down-regulated by the Auxin, BR and JA treatments in the roots, while its expression was up-regulated by all the hormone treatments, except JA, in the shoots (Figure 7J). The expression of *LOC_Os11g32750* in the roots was significantly up-regulated by all six hormones treatments, while in the shoots, it was up-regulated by the GA and Auxin treatments and down-regulated by the ABA, BR and JA treatments (Figure 7K). The expression of *LOC_Os11g32810* was significantly up-regulated in the shoots by all six hormones treatments except JA, while in roots, it was significantly up-regulated by the ABA, GA, Auxin and JA treatments (Figure 7L). Overall, the 12 predicted candidate genes all responded to different hormone treatments, suggesting that they may also contribute to the hormone homeostasis in seeds, which is important for dormancy/germination.

## 3. Discussion

### 3.1. Phenotypic Variation of Seed Dormancy

Seed dormancy is influenced by genetic factors, with significant variation in different subspecies [34,80,81,82]. Our results showed that the mean GR of *indica* (69.0%) was significantly higher than that of *japonica* rice (58.9%) in the WDB control, and it was slightly higher under both the DH and GA treatments (Table 1). Previous studies also found that *indica* showed non-deep dormancy [34,82]. Compared to other studies, the average GR of our *indica* subspecies was lower. This was caused by the low GR of AUS accessions (Table S6). AUS is a unique ecotype with narrow geographical distribution and has less genetic similarity with other *indica* subpopulations. AUS usually has much lower GR than other *indica* subpopulations do (Table S6), which can be exploited to identify genes for seed dormancy.

Freshly harvested rice seeds have a low GR due to post-harvest dormancy, which usually disappears after dry storage [20,83]. In rice production, some physical measures such as DH treatment, which disrupts seed structure (i.e., seed coat and perisperm) [20,84,85], and chemical measures such as GA treatment, which mediates α-amylase synthesis and weakens the endosperm cap [86,87], are commonly used to break dormancy before sowing to promote germination. Therefore, it is expected that all accessions have a higher GR under the DH and GA treatments (Figure 2, Table 1). We found that the GA treatment was more effective than the DH treatment (Table 1). It is important for identification of QTLs/genes for seed dormancy/germination to conduct germination assay in a proper environment. 

### 3.2. GWAS of Rice Seed Dormancy

GWAS utilizes the accumulated historical recombination and has higher mapping resolution than the conventional linkage mapping using biparental populations do [11,88,89]. In recent years, GWAS in crops has gained popularity, since it can be applied to populations of accession and/or breeding lines and is a component of germplasm characterization and breeding programs [11]. In this study, 195 rice accessions from 40 different countries constitute a genetically diverse population for GWAS, which are clearly grouped into *indica* (XI-1, XI-2, XI-3 and AUS), *japonica* (GJ-tmp, GJ-trp, GJ-sbtrp and Basmati) subpopulations (Figure 3B, Table S1); this population with rich genomic diversity and great phenotypic variation would be more favorable for studying the genetic architecture of complex traits in rice [90,91]. Here, we employed resequencing-based GWAS to identify relevant SNP markers to determine the genetic variation in seed dormancy. As we showed, the high density of SNP markers (3 SNPs per kb) allowed the high resolution mapping of genomic regions controlling seed dormancy/germination-related traits through GWAS. In our GWAS, five, nineteen and sixteen QTLs were identified using the MLM-PCA-K model under WDB control, DH and GA treatments, respectively (Figure 4, Table S2). We found that more QTLs were detected under the dormancy breaking treatments (DH and GA). More association signals were found using post-ripening (dormancy breaking) seeds than those found using fresh seeds (dormancy) in a previous GWAS analysis [34]. Also, more co-localized QTLs were found between the two dormancy breaking treatments, indicating more QTLs/genes controlling seed dormancy were identifiable under the dormancy breaking conditions.

The parameters (traits) used to determine seed dormancy in rice are seed GR, GI, germination potential and germination uniformity, among which, seed GR is the most commonly used one [34,42,82,92]. In our study, QTLs for dormancy using GR as the criterion were distributed on all chromosomes, except chromosomes 7 and 12, with chromosomes 4 and 11 containing more QTLs (Figure 4 and Figure 5), which is in agreement with the general distribution pattern of rice seed dormancy QTLs, especially those identified by GWAS [27,34,35]. Ten of the thirty QTLs identified in this study overlapped with QTLs previously identified by linkage mapping or GWAS (Table 2). *qGR1-1* (2.9–3.1 Mb) on chromosome 1 overlapped with *qDOM1.1* [28]. *qGR1-2* (23.8–24.0 Mb) on chromosome 1 was physically close to the *qSD-1-1* [41]. *qGR6-2* (13.0–13.2 Mb) on chromosome 6 was co-localized with both *qDOM6.2* [28] and *ARS6.3* for after-ripening seed germination [34]. *qGR8* (7.9–8.2 Mb) on chromosome 8 was in close proximity to *qDOM8.1* [28]. The location of *qGR9-3* at 11.2–11.4 Mb on chromosome 9 was comparable to that of *qDOR-9-1* (detected intact seed or de-hulled seed 30 days after heading [42]. *qGR11-1* (9.6–9.8 Mb) on chromosome 11 was in close proximity to *seq-rs4917* [2] and *qSD-11* [29]. *qGR11-4* (19.2–19.4 Mb) on chromosome 11 was co-localized with *ARS11.4* [34] and *qDOR-11-3* [42]. *qGR11-5* (20.5–20.7 Mb) on chromosome 11 was close to the QTL *qDOR-11-4* [42]. Both *qGR11-6* (23.8–24.0 Mb) and *qGR11-7* (24.0–24.2 Mb) on chromosome 11 were positioned close to *qDOR-11-6* [42]. The population composition, seed development stage and testing environment are major factors contributing to the differences in the mapping results [34,42,93,94]. Nevertheless, the existence of multiple QTLs in all the studies suggests that rice seed dormancy is a complex trait controlled by many genes and is greatly affected by the environmental conditions.

In our study, we found only a few commonly detected QTLs for the different treatments. Only one QTL, *qGR11-4*, was identified in all the three treatments, and the total number of common QTLs for any of two treatments was three. This was surprising, since the phenotypic correlations between the three environments were positive and high. The low number of QTLs identified under the WDB control could partially explain the low number of common QTLs between the WDB control and the other two treatments. However, it could not explain why there were only two common QTLs between DH and GA. It is very likely that different pathways were involved in the three treatments.

### 3.3. Candidate Genes for Seed Dormancy

Five important QTLs explained appreciable variation in GR were chosen for predicting candidate genes, namely *qGR3*, *qGR4-4*, *qGR4-5*, *qGR8* and *qGR11-4* (Table 2). Five high-confidence candidate genes, including *LOC_Os03g50050*, *LOC_Os03g50110*, *LOC_Os03g50130*, *LOC_Os03g50140* and *LOC_Os03g50160,* were identified for *qGR3* on chromosome 3. *LOC_Os03g50050* encodes an F-box domain-containing protein that is highly expressed in the ovaries and endosperms at early stages of seed development (Figure 6A) and is significantly up-regulated by ABA, GA and JA treatments (Figure 7A). *OsFbx352*, a homolog of *LOC_Os03g50050,* was reported to be a regulator of the glucose-induced seed germination inhibition by reducing the level of endogenous ABA [45]. In *Arabidopsis*, several F-box proteins were reported to regulate seed dormancy/germination by mediating ABA [46,47] or GA [44]. Therefore, *LOC_Os03g50050* is most likely to be involved in seed dormancy by regulating the ABA/GA metabolism. *LOC_O03g50110* encodes a GEBP transcription factor expressed in the inflorescences and embryos (Figure 6B) and is significantly regulated by multiple hormone treatments, especially in roots treated with ABA, GA, Auxin, CTK and JA treatments (Figure 7B). The GEBP transcription factor family has been reported to function in the CTK hormone pathway [49,50], and CTK has a promotive effect on seed germination by antagonizing ABA, the level of which is generally lower in dry seeds, but increases during germination, specifically by down-regulating ABA-Insensitive 5 (*ABI5*) transcription and inducing ABI5 protein degradation to promote seed germination in *Arabidopsis thaliana* [51,52]. *Arabidopsis* CTK receptor mutants had lower levels of dormancy compared to those of the wild type [48]. Therefore, *LOC_O03g50110* is most likely to contribute to seed dormancy by controlling the CTK/ABA cross-talk in rice. *LOC_Os03g50130* encodes the microsomal glutathione S-transferase (GST) 3 protein expressed in the ovaries (Figure 6C) and has a significantly higher expression at 24 h of imbibition (Table 3) and is very responsive to multiple hormones, such as ABA, BR, CTK and JA treatments (Figure 7C). Plant GST family genes play important roles in abiotic stresses and affect seed germination. The *atgstu7* and *atgstu17* mutants accumulated higher levels of GSH and ABA and exhibited low sensitivity to ABA during seed germination in *Arabidopsis* [53,56]. The heterologous expression of *OsGSTU4* in *Arabidopsis* reduced the sensitivity to ABA and the levels of reactive oxygen species (ROS) and increased seed germination under high levels of salt and ROS stress in transgenic *Arabidopsis* [54]. The overexpression of *AtGSTU19* increased the rate of seed germination under salt/drought stress in *Arabidopsis* [55]. Therefore, it is speculated that *LOC_Os03g50130* may regulate seed dormancy/germination through influencing GSH/ROS-mediated hormone homeostasis. *LOC_Os03g50140* and *LOC_Os03g50160* both encode plastocyanin (PCY)-like domain containing proteins, which are highly expressed during mature seed development (up to approximately 2–145 times) (Figure 6D,E), have significantly higher expression levels at 12 h and 24 h of imbibition (Table 3) and are greatly affected by different hormones (Figure 7D,E). Previous studies have proposed that the Phytochrome A (phyA)-Phytochrome Interacting Factor 1 (PIF1)-miR408-PCY cascade regulates light-dependent germination in *Arabidopsis* [57]. PCY is highly conserved and evolved specifically as a target protein of miR408 in seed plants [57]. *LOC_Os03g50140* and *LOC_Os03g50160* are likely to be target proteins of miR408 and/or other miRNAs and contribute to seed germination through regulating the hormone levels. *LOC_Os04g18200* and *LOC_Os04g18380* were identified for *qGR4-4*. *LOC_Os04g18200* encodes the dihydrodipicolinate synthase (DHDPS), is significantly down-regulated at 12 h and 24 h imbibition (Table 3) and expresses in mature inflorescences, ovaries and embryos (Figure 6F), and it is significantly induced by various hormones, especially ABA, GA Auxin and CTK (Figure 7F). In higher plants, the reaction catalyzed by DHDPS is the first step in lysine (Lys) biosynthesis and is regulated by Lys through feedback inhibition [61]. In *Arabidopsis*, a high seed Lys content was found to be a major reason for delayed germination [59], and similar results were also observed in soybean and canola seeds [58,60]. These results suggested that Lys accumulation is negatively correlated with seed germination and that *LOC_Os04g18200* may play a pivotal role in seed dormancy/germination through the Lys synthesis pathway. *LOC_Os04g18380* is annotated as a cytochrome P450 protein. In *Arabidopsis*, P450 monooxygenase 707A (*CYP707A*) and *CYP707A2* encode the ABA catabolic enzyme 8'-hydroxylases and play an important role in regulating the seed ABA level, and thus dormancy [62,63]. Therefore, it can be speculated that *LOC_Os04g18380* is likely to play a significant role in the control of the seed ABA level, and therefore dormancy and germination. Two high-confidence candidate genes were identified for *qGR4-5*. *LOC_Os04g18650* encodes an AP2 domain-containing protein expressed in the leaves, anthers, ovaries, and inflorescences during ripening (Figure 6G) and is significantly induced by ABA, BR and CTK (Figure 7G). Some of the AP2 domain containing proteins play a dual role in the regulation of ABA and GA biosynthesis and participate in seed dormancy and germination [64,65,66]. Therefore, *LOC_Os04g18650* may regulate seed dormancy by regulating ABA and GA biosynthesis as well. *LOC_Os04g18790* encodes an F-box domain-containing protein highly expressed in the inflorescences, ovaries and embryos at the ripening stage (Figure 6H) and is induced by ABA, GA, Auxin, BR, CTK and JA (Figure 7H). Therefore, similar to *LOC_Os03g50050*, a candidate gene for *qGR3*, *LOC_Os04g18790* may affect seed dormancy by affecting the balance of multiple hormones in seeds. For *qGR8* identified under both DH and GA treatments, a high-confidence candidate gene is *LOC_Os08g13640*, which encodes ankyrin, is highly expressed in the inflorescences, ovaries and embryos during the seed development stage (Figure 6I), is down-regulated after 12 h and 24 h of imbibition (Table 3) and is especially responsive to ABA (Figure 7I). In plants, ankyrin proteins are extensively involved in biotic and abiotic stresses. For instance, the *Arabidopsis itn1* (increased tolerance to NaCl 1) mutant partially impairs the ABA signaling transduction pathway, leading to enhanced salt tolerance by reducing ROS accumulation under salt stress [67]. The heterologous expression of the *soybean GmANK114* gene in *Arabidopsis* resulted in a higher GR under drought and salt stresses [68]. Therefore, *LOC_Os08g13640* may be involved in seed germination by regulating the seed endogenous ABA level.

*LOC_Os11g32650*, *LOC_Os11g32750* and *LOC_Os11g32810* were high-confidence candidate genes for *qGR11-4* detected under all the three treatments. *LOC_Os11g32650* encodes the CHS expressed in the paddy hull (lemma and palea) and embryos (Figure 6J), has a significantly higher expression level at 12 h of imbibition (Table 3) and is regulated by JA treatment (Figure 7J). CHS is the first key enzyme of the flavonoid pathway and plays a central role in the pigmentation of the plant organs, seed germination and biotic stress [70,72]. In *Arabidopsis*, the *BANYULS* (*Ban*) functions as a negative regulator of the flavonoid biosynthesis that prevents the accumulation of pigments in the seed coat during early embryo genesis; *ban* mutants exhibit reduced germination and accumulated pigments in the seed coat [69]. In tobacco, *EaCHS1* allows plantlets to tolerate salinity stress by maintaining ROS homeostasis, and the overexpression of *EaCHS1* improves the resistance to salinity stress by altering the accumulation of flavonoids during seed germination [71]. Therefore, it is possible that *LOC_O11g32650* contributes to seed dormancy/germination via the flavonoid pathway in rice. Whether hormones affect CHS biogenesis and/or signaling during the transition from dormancy to germination will be a pertinent topic for future study. *LOC_Os11g32750* encodes an NUDIX family hydrolase. The *NUDIX* family genes participate in various cellular and metabolic processes and affects seed germination in several species [73,74]. For instance, the changes in nicotinamide adenine dinucleotide (NADH) pyrophosphate hydrolase activity conferred by nudix hydrolase 7 (*NUDT7*) affect seed dormancy mainly by altering the ABA and ROS levels in *Arabidopsis* seeds, and the loss of function of *AtNUDT7* resulted in high levels of ABA accumulation and reduced the germination rate [73]. Overexpression of the *PpNUDX8* from *Pessica* affects the NADH contents in tobacco, resulting in a reduced ABA content in tobacco seedlings and reduced the sensitivity to ABA during germination [74]. *LOC_Os11g32750* has a low expression level in the inflorescences, ovaries, embryos and endosperms (Figure 6K), is significantly up-regulated at both 12 and 24 h of imbibition (Table 3) and is significantly regulated by ABA, GA, Auxin, BR and JA treatments (Figure 7K). Therefore, *LOC_Os11g32750* may play a role in regulating seed dormancy/germination by regulating the NADH-dependent ABA synthesis pathway. *LOC_Os11g32810* encodes an LRR family protein. LRR proteins involved in seed dormancy were reported in several species [75,76,77]. The *atrlk7* mutants cause delayed seed germination, whereas their overexpression plants cause an earlier germination phenotype in *Arabidopsis* [75]. The overexpression of Floral Organ Number 1 (*FON1*) delayed seed germination and increased ABA sensitivity in rice [76]. The heterologous expression of *PnLRR-RLK2* (*Pohlia nutans*) in *Arabidopsis* increased the tolerance to salt and ABA during seed germination and GR [77]. *LOC_Os11g32810* is constitutively expressed at different growth stages with a higher expression level in the inflorescences and ovaries (Figure 6L), is significantly down-regulated at 24 h of imbibition (Table 3) and is significantly induced by multiple hormones (Figure 7L). Therefore, it is highly likely that *LOC_Os11g32810* is involved in seed dormancy/germination by receiving ABA signals and transmitting hormone signals to downstream targets.

## 4. Materials and Methods

### 4.1. Plant Material and Growth Conditions for Multiplication

The association mapping population consisted of 195 rice accessions from the Chang Genetic Resources Center at the International Rice Research Institute (IRRI). Of these, 172 accessions were re-sequenced by the 3K Rice Genome (3K-RG) [91], and the other 23 accessions were re-sequenced by our team. All accessions belong to the *indica* (72) and *japonica* (123) subspecies, with the *indica* accessions being grouped into Xian/Indica-1 (XI-1), Xian/Indica-2 (XI-2), Xian/Indica-3 (XI-3) and AUS subpopulations, and the *japonica* accessions being grouped into Geng/Japonica-temperate (GJ-tmp), Geng/Japonica-tropical (GJ-sbtrp), Geng/Japonica-subtropical (GJ-trp) and Basmati subgroups (Table S1). 

All accessions were grown in the paddy fields of the Agricultural Genomics Research Institute in Shenzhen, Chinese Academy of Agricultural Sciences (CAAS-AGIS, Shenzhen, China), on June 2020. The 25-day-old seedlings were transplanted to the experimental field. The plants were grown with 20 cm between the plants within a row and 20 cm between the rows and managed according to local field management practices. The heading time of each accession was recorded when the first panicle emerged. All spikes were harvested approximately 30–40 days after heading [95] and stored at 4 °C immediately after being harvested to maintain seed freshness for the germination evaluation.

### 4.2. Assessment of Seed Germination 

The seeds were removed from spikes and pooled for the germination test. The three treatments are as follows. For without the dormancy breaking (WDB) control, seeds stored at 4 °C were used directly for the germination test using sterile water, for the dry heating (DH) treatment, seeds were dried in the oven at 45 °C for 3 days before germination test, and for the gibberellic acid (GA) treatment, seeds stored at 4 °C were tested for germination in 5 μM GA solution. A randomized complete block design with three replicates was used to layout the experiment for each treatment. For each replicate, 150 seeds per accession were evenly spread in Petri dishes (20 cm × 15 cm × 3 cm) containing 35 mL sterile water or 5 μM GA solution moistened with filter paper and cultured in a constant temperature light incubator at 30 °C with 16 h light and 8 h dark and 80% relative humidity. The seeds with radicles longer than 2 mm were regarded as germinated seeds after 7 days of incubation [96]. The GR was calculated as (the number of germinated seeds/the total number of seeds) × 100%. The mean GR of the three replicates was used for GWAS. To facilitate graphical presentation, a scale of 1–8, with a score of 1 for the germination rate (GR) 0–12.5%, 2 for 12.5–25%, 3 for 25–37.5%, 4 for 37.5–50%, 5 for 50–62.5%, 6 for 62.5–75%, 7 for 75–87.5% and 8 for 87.5–100%, was adopted to group the accessions. With this scoring, the seed dormancy types were classified as deep (score 1, 2 and 3), intermediate (score 4, 5 and 6) and non-deep (score 7 and 8) ones [39]. 

### 4.3. Genotyping 

Total genomic DNA was extracted from young leaves of 23 accessions with sequence information according to the cetyl trimethyl ammonium bromide (CTAB) method [97]. Re-sequencing was conducted by the Berry Genomics Corporation (https://www.berrygenomics.com/, accessed on 1 June 2019) (Beijing, China) using an Illumina HiSeq 2000 (PE150). The average sequencing depth of each accession genome was 50×. Analysis of the raw reads to obtain SNPs followed the same procedure used in the 3K-RG Project [91]. Reads were aligned to the Nipponbare RefSeq (IRGSP-1.0) using BWA-MEM (release 0.7.10). The mapped reads were then sorted, and duplicates were removed by Picard tools (http://broadinstitute.github.io/picard/, accessed on 1 June 2019). The variants for each accession were called by the GATK best practices (release 3.2-2). The genotype data for the other 172 accessions were obtained from the 3K-RG Project. The average sequencing depth was 11.5×, and the average mapping coverage was 92% [91].

### 4.4. Population Structure Analysis 

Principal component analysis (PCA) and neighbor-joining (NJ) tree analysis were used to investigate the population stratification of the association panel. The PCA plot was generated by Pot3D and Satterplot3d packages based on R v3.2.2 (https://www.r-project.org/, accessed on 1 March 2021). Nei’s genetic distance (1972) was calculated and used for unrooted phylogeny reconstruction based on the NJ method as implemented by PowerMarker v2.3.5 software [98], and the NJ tree was visualized using Figtree (http://tree.bio.ed.ac.uk/software/figtree/, accessed on 1 March 2021).

### 4.5. Linkage Disequilibrium Analysis

The linkage disequilibrium (LD) analysis was performed using a set of filtered SNP markers (MAF ≤ 5%) and the LD function in TASSEL v.5.0 [99]. The squared correlations of allele frequencies between marker pairs (*r^2^*) were used to estimate the LD. Using the rapid simulation method in Tassel v5.0, the full matrix and sliding window were used to calculate the LD between the markers, with the number of Permutations being set to 1000. The makers with a significant LD were identified based on *p* < 0.001. The LD decay plot was generated by plotting the *r^2^* against the physical distance between the marker loci. The *r^2^* value at the 95th percentile was used as the threshold to estimate the LD decay, and the intersection with the LD decay curve was used as the LD decay distance. 

### 4.6. Genome-Wide Association Analysis

SNPs with a MAF ≤ 5% or a missing data rate ≥ 10%missing ratio were filtered out using Tassel v5.0 software, and the remaining 1,120,223 SNPs were subsequently employed for GWAS. GWAS analysis was conducted using the mixed linear model (MLM) with PCA and K as covariates. The significance threshold was set to *p* ≤ 1.0^−4^. The GWAS results were visualized as Manhattan and Quantile–Quantile (QQ) plots using the CMplot software package (https://github.com/YinLiLin/R-CMplot, accessed on 1 March 2021) of R v3.2.2 (https://www.r-project.org/, accessed on 1 March 2021). Based on LD decay analysis, significant makers within a 200 kb interval (±100 kb) of the peak SNP were integrated as a single QTL.

### 4.7. Candidate Gene Analysis

The candidate genes for QTLs identified in two or three treatments were identified. The following steps were taken to identify high-confidence candidate genes from the genes in a QTL region extracted using the MSU Rice Genome Annotation Database (http://rice.plantbiology.msu.edu, accessed on 1 September 2022). First, genes annotated as expressed proteins, hypothetical proteins, transposon proteins and retrotransposon proteins were filtered. Second, genes without non-synonymous mutations in the coding region and variants in the promoter region (−2000 bp from the start codon) were removed. Third, a careful literature search was conducted to find the reported functions of the remaining genes and their homologous genes. At the same time, the expression patterns of these genes in imbibed rice seed embryos were analyzed using the EBI Expression Atlas Database (https://www.ebi.ac.uk/gxa/home, accessed on 1 November 2022) [43]. The genes with significant differential expression between the dry and imbibed seeds were kept. Fourth, the gene lists formed of the literature information and expression during imbibition were combined to form the high-confidence candidate genes.

To collect more information about the candidate genes, datasets of two historical studies were downloaded from the Rice Expression Profile Database (https://ricexpro.dna.affrc.go.jp/, accessed on 1 November 2022) [78] and used to analyze their expression in different tissues/organs throughout entire growth period in the field and the responses to treatments of various plant hormones at the seedling stage. The expression levels in roots in the vegetative stage and untreated seedlings (0 h) were used as controls for analysis, respectively.

## 5. Conclusions

Five genes with natural variants were reported to affect the seed dormancy trait in rice, which can be used in marker-assisted breeding to increase efficiency. The studies on these genes also greatly advanced our understanding of the molecular mechanisms of seed dormancy in rice. More knowledge on the functions of individual genes and their interactions will further improve the efficiency of molecular breeding. Therefore, the identification and functional characterization of more genes is essential. Using a population of 195 diverse re-sequenced rice accessions, the present study identified 30 QTLs that explained 7.3–20.4% of the GR variation under three treatments. Our results further proved that seed dormancy in rice is a highly complex trait controlled by many genes. This implies that successful molecular breeding requires the use of multiple genes from multiple biological pathways, and a clear understanding of the interactions between these genes and pathways is very important. Although false positives cannot be completely ruled out since a less stringent threshold *p* value was used to reduce the potential false negatives, our result that the majority (20) of the identified QTLs (30) are not reported previously does indicate that GWAS using a more diverse population with high-quality sequence information is a powerful method for dissecting the genetic basis of complex traits in rice. Only four QTLs were identified under two or three treatments, although the phenotypic correlations between the treatments were positive and strong. This suggests that phenotypic correlation between two environments for a complex trait is not necessarily a good indicator of the existence of common QTLs/genes in the two environments. Different pathways are likely to play more important roles in different environments. The 13 high-confidence candidate genes predicted for the four QTLs identified in more than one treatment and *qGR3* with a large effect are all directly or indirectly related to the biosynthesis and metabolism of ABA, GA or CTK, although their responses, in terms of expression, to these hormones were not used as selection criteria. This is consistent with the established key roles of these hormones in seed dormancy in crops in general, especially in rice. These candidate genes are important targets for functional validation in future studies to decipher the molecular mechanisms of seed dormancy in rice and provide valuable genes for molecular breeding. Our results also proved that the AUS ecotype has stronger dormancy. Therefore, mining for genes/alleles with strong dormancy traits using AUS germplasms will be more fruitful. The accessions with strong and moderate dormancy identified in this study can be used in breeding for developing varieties with a high dormancy level to reduce the damage of PHS, which often occurs in *indica* rice production.

## Figures and Tables

**Table 1:** Descriptive statistics of seed dormancy measured as germination rate (%) under three treatments.

Trait	Seed Germination (%)			
Treatment	Terms	*Japonica*	*Indica*	Whole
WDB control	Min	0.0	0.0	0.0
	Max	94.5	96.7	96.7
	Mean	58.9	69.0	62.4
	SD	25.0	26.7	26.0
	CV (%)	42.4	38.6	41.7
DH	Min	3.1	0.0	0.0
	Max	98.0	100.0	100.0
	Mean	67.6	71.2	69.0
	SD	23.2	32.8	27.2
	CV (%)	34.3	46.1	39.5
GA	Min	2.4	2.7	2.4
	Max	98.5	98.5	98.5
	Mean	73.0	74.5	73.6
	SD	21.8	29.2	24.7
	CV (%)	29.9	39.1	33.6

WDB control, without dormancy breaking as a control treatment; DH, dry heating treatment; GA, gibberellic acid treatment; SD, standard deviation; CV, coefficients of variation.

## Data Availability

Data are contained within the article or Supplementary Materials.

## Supplementary Materials

The following supporting information can be downloaded at: https://www.mdpi.com/article/10.3390/ijms24076117/s1.
